# Ally, adversary, or arbitrator? The context-dependent role of eosinophils in vaccination for respiratory viruses and subsequent breakthrough infections

**DOI:** 10.1093/jleuko/qiae010

**Published:** 2024-01-30

**Authors:** Lauren A Chang, Michael Schotsaert

**Affiliations:** Graduate School of Biomedical Sciences, Icahn School of Medicine at Mount Sinai, One Gustave L. Levy Place, New York, NY 10029, United States; Department of Microbiology, Icahn School of Medicine at Mount Sinai, One Gustave L. Levy Place, Box 1124, New York, NY 10029, United States; Global Health and Emerging Pathogens Institute, Icahn School of Medicine at Mount Sinai, One Gustave L. Levy Place, Box 1124, New York, NY 10029, United States; Department of Microbiology, Icahn School of Medicine at Mount Sinai, One Gustave L. Levy Place, Box 1124, New York, NY 10029, United States; Global Health and Emerging Pathogens Institute, Icahn School of Medicine at Mount Sinai, One Gustave L. Levy Place, Box 1124, New York, NY 10029, United States; Marc and Jennifer Lipschultz Precision Immunology Institute, Icahn School of Medicine at Mount Sinai, 1425 Madison Avenue, Box 1630, New York, NY 10029, United States; Icahn Genomics Institute, Icahn School of Medicine at Mount Sinai, One Gustave L. Levy Place, New York, NY 10029, United States

**Keywords:** eosinophils, respiratory viruses, vaccines, viral infection

## Abstract

Eosinophils are a critical type of immune cell and central players in type 2 immunity. Existing literature suggests that eosinophils also can play a role in host antiviral responses, typically type 1 immune events, against multiple respiratory viruses, both directly through release of antiviral mediators and indirectly through activation of other effector cell types. One way to prime host immune responses toward effective antiviral responses is through vaccination, where typically a type 1–skewed immunity is desirable in the context of intracellular pathogens like respiratory viruses. In the realm of breakthrough respiratory viral infection in vaccinated hosts, an event in which virus can still establish productive infection despite preexisting immunity, eosinophils are most prominently known for their link to vaccine-associated enhanced respiratory disease upon natural respiratory syncytial virus infection. This was observed in a pediatric cohort during the 1960s following vaccination with formalin-inactivated respiratory syncytial virus. More recent research has unveiled additional roles of the eosinophil in respiratory viral infection and breakthrough infection. The specific contribution of eosinophils to the quality of vaccine responses, vaccine efficacy, and antiviral responses to infection in vaccinated hosts remains largely unexplored, especially regarding their potential roles in protection. On the basis of current findings, we will speculate upon the suggested function of eosinophils and consider the many potential ways by which eosinophils may exert protective and pathological effects in breakthrough infections. We will also discuss how to balance vaccine efficacy with eosinophil-related risks, as well as the use of eosinophils and their products as potential biomarkers of vaccine efficacy or adverse events.

## Teaser

Review on the described and postulated roles for eosinophils in vaccination for respiratory viruses, as well as how eosinophils may impact vaccine responses and the outcome of breakthrough infections.

## 1. Introduction

Vaccines are one of the most important public health measures and medical interventions for preventing severe disease, hospitalization, and death for numerous infectious diseases.^1,2^ Vaccines limit the incidence rate of both seasonal and pandemic respiratory viral infections, such as influenza virus and severe acute respiratory syndrome coronavirus 2 (SARS-CoV-2).^3–5^ Currently, several types of vaccines and adjuvants exist for respiratory viruses with different inherent immunological properties, allowing vaccine developers to tune immunogenicity, potency, and the very nature of the immune response elicited.^6–9^ As summarized by Annunziato et al.,^10^ the 3 major types of immunity are as follows: type 1 immunity for control of intracellular pathogens, type 2 immunity for control of extracellular pathogens and venoms, and type 3 immunity for control of extracellular bacteria and fungi. Respiratory viruses are intracellular pathogens; therefore, eliciting type 1 immunity is generally desirable.^11–13^ Some hallmarks of type 1–skewed adaptive immunity include generation of neutralizing antibody isotypes such as IgG2a/c in mice or IgG1 and IgG3 in humans, IFN-γ–secreting CD4 T cells, and cytotoxic CD8 T cells.^10,11^ Collectively, these factors favor the clearance of infected cells and limit further viral replication.

Nonetheless, even when potent type 1 immunity is elicited, such as in the case of mRNA vaccinations, breakthrough infections can still occur: neutralizing antibody titers and antigen-specific lymphocytes in circulation may wane over time, sufficient degrees of viral antigenic mutation may result in immune escape, or when both of these occur concurrently.^14–21^ Mediators of type 1–biased immunity, such as preexisting memory IFN-γ^+^ CD8 T cells, can effectively clear viral infection by directly killing infected cells while inducing an antiviral state in the infected host tissue during infection.^22–24^ However, depending on the nature of the priming event, type 2–biased responses may arise during secondary antigenic exposure during breakthrough infection and result in adverse secondary outcomes. A prominent example of how a type 2 response during breakthrough infection can result in pathology and worse host outcomes is well documented in the formalin-inactivated respiratory syncytial virus (FI-RSV) vaccine study in the 1960s.^25^ Respiratory syncytial virus (RSV)–naive infants that received the FI-RSV vaccine adjuvanted with alum had more severe disease upon natural RSV infection compared to infants that did not receive the vaccine.^25–29^ This specific phenomenon was termed vaccine-associated enhanced respiratory disease (VAERD), and several hallmarks have been identified: enhanced type 2 cytokine production (IL-4, IL-5, IL-13), bronchial cuffing and increased lung pathology, mucus hypersecretion and goblet cell hyperplasia, increased host morbidity, and lung eosinophilia.^30–36^ VAERD has also been described for other respiratory virus–vaccine pairs, such as for SARS-CoV, SARS-CoV-2, and influenza.^37–43^ Nonpathological lung eosinophil recruitment has also been observed in mouse models of influenza breakthrough infection.^44–46^

The exact function of recruited lung eosinophils in the context of breakthrough infection, both pathological like VAERD or nonpathological, is not fully understood. Eosinophils are important cellular mediators of type 2 immunity that are implicated in antihelminth responses and in the pathogenesis of allergy and asthma, so their recruitment to the lung during a type 1 immune event like viral infection seems at odds with previously described roles.^47–55^ However, there are multiple studies in preclinical animal models indicating that eosinophils can contribute to antiviral responses and that the infiltration of eosinophils into the lung can correlate with protection rather than pathology in a way that is distinct from VAERD during both primary respiratory viral infection and breakthrough infection.^44–46,56–65^

In this review, we aim to synthesize currently published data on the role of eosinophils in vaccines for respiratory viral infection and postulate how eosinophils may mediate protection, pathology, or both upon subsequent breakthrough infection. We discuss balancing vaccine efficacy with eosinophil-related risks and eosinophils as biomarkers for vaccine efficacy or adverse events.

## 2. Vaccines for respiratory viruses, target responses, and the impacts of immune skewing

Before we discuss the role of eosinophils in vaccination and breakthrough infection, we must first understand the immunological backdrop set by the vaccination itself, including the purpose of vaccines for respiratory viral infections, the type of immune responses required for effective vaccine protection, and how to leverage polarization of vaccine responses while generating protective immunity. Respiratory viral infections such as influenza, SARS-CoV-2, RSV, and rhinovirus are one of the leading causes of illness and death across the world.^66^ Effective vaccines against respiratory viruses reduce disease severity through the immunological protection of the host.^1,2^ In infection, correlates of protection against respiratory viruses are highly associated with induction of a type 1 response, including neutralizing antibodies, particularly mucosal antibodies such as secretory IgG and IgA, as well as CD4 T helper 1 (Th1) memory cells and virus-specific cytotoxic CD8 T cells.^67–72^ As such, vaccines that effectively induce a type 1 immune response are generally favorable to provide protection from respiratory viral infection.^10,12,13^

However, while type 1 immunity plays a major role in protection, type 2 immunity has also been shown to play a role in influencing outcomes following primary viral infection. Type 2 cytokines such as IL-4 and IL-13 can promote B-cell proliferation and class switching alongside robust production of antibodies but can also block the differentiation of Th1 CD4 T cells.^12^ Additionally, the ability for type 2 immune mediators to restrain type 1–skewed inflammation is especially important after viral clearance in mitigating excess inflammation, promoting tissue repair and regeneration, and restoring barrier homeostasis.^73^ ST2^+^ regulatory T cells (Tregs) can induce the production of amphiregulin and protect the lung from tissue damage after influenza infection.^74^ Additionally, a defective type 2 response may result in adverse outcomes following viral infection. For example, IL-4^−/−^ mice are more susceptible to lung injury following *Streptococcus pneumoniae*/influenza coinfection due to increases in both gasdermin D–induced pyroptosis of macrophages and higher concentrations of inflammatory cytokines, neither of which were observed in wild-type mice with intact IL-4 production.^75^ Furthermore, administration of recombinant IL-4 to coinfected IL-4^−/−^ mice reduced IL-6, TNF-α, and IL-1β levels and the degree of lung injury.^75^

Despite the potentially protective function of type 2 immunity in response to respiratory viral infections, the deleterious effects of excessive type 2 skewing are well documented. Notably, rhinovirus or RSV infection, particularly in young children, can result in virus-induced asthma.^76–78^ IL-4 treatment or constitutive overexpression delayed clearance of influenza virus and RSV in mouse models.^79,80^ CD4 T helper 2 (Th2) cell immunity elicited by FI-RSV vaccination was shown to be a major driver of airway hyperresponsiveness and mucus hypersecretion in a mouse model of VAERD for RSV.^33^ Beyond the lung, coinfection studies with herpes simplex virus (HSV) 2 infection and helminths showed that excessive eosinophilia in the female genital tract correlated with worse viral pathology, even if no direct damage from the helminth was involved.^81^ Thus, it is thought that eliciting strong, type 1–dominated responses during vaccination with minimal type 2 responses is tantamount to conferring potent antiviral immunity, especially in organs such as the lung, where skewing of host immunity toward type 2 can have adverse outcomes such as VAERD or asthma. However, the complete absence of type 2 immunity can result in adverse outcomes as well, such as excessive immunopathology. Therefore, vaccination should aim to capture the benefits of both type 1 and type 2 immunity, balancing the cytolytic, proinflammatory effects of type 1 immunity with the proresolving, wound-healing effects of type 2 immunity.

There is growing evidence that induction of a balanced type 1/type 2 response after viral vaccination can be beneficial.^82–90^ A head-to-head comparison of mice vaccinated with recombinant influenza hemagglutinin (HA) protein adjuvanted with type 2–skewing alum, type 1–skewing phosphorylated hexa-acyl disaccharide (PHAD), or bacterial enzymatic combinatorial chemistry (BECC) molecules 438 and 470 revealed that BECC molecules conferred a balanced type 1/type 2 response and resulted in superior protection against weight loss and lung pathology, as well as greater reductions in viral titers after vaccine-matched viral challenge compared to other groups, including the more type 1–skewed PHAD-adjuvanted group.^89^ Vaccination of mice with prefusion-stabilized RSV fusion (F) protein with adjuvants that conferred a more balanced Th1/Th2 response resulted in greater neutralization capacity compared to other vaccine–adjuvant pairs.^90^ More mechanistic studies directly comparing type 1–skewed, type 2–skewed, and a balanced type 1/type 2 response in the context of vaccination and viral immunity, especially beyond the acute infection time frame and in the respiratory tract, will be necessary to understand the impact of immune polarization on long-term host outcomes. Additionally, it will be important to understand how to temporally regulate type 2 responses to occur after acute infection to maximize tissue repair capabilities but minimize inhibition of the early type 1 responses needed to rapidly clear virus.

Vaccines have been proven to be highly effective at reducing disease severity, at times generating sterilizing immunity and complete prevention of symptomatic disease. This variability can be due to vaccine platform, adjuvant inclusion, and the target virus itself. Additionally, maintaining protection throughout the entire host life span postvaccination is highly unlikely due to host-intrinsic baseline differences in immunity (age, sex, comorbidities, genetics, environment), natural waning of protective antibodies, immunosenescence, and viral antigenic escape from antibodies and memory lymphocytes. Therefore, breakthrough infection is a frequent phenomenon, especially for constantly evolving respiratory viruses such as influenza. As such, when designing vaccines, we must be intentional in the selection of adjuvants and vaccine platforms to appropriately modulate innate and adaptive immune responses both after vaccination and after breakthrough infection.^91–97^ For the purpose of this review, we will focus on parenteral vaccines, although intranasal and other mucosal vaccines are gaining prominence.

## 3. Eosinophils during breakthrough infection in hosts vaccinated against respiratory viruses

A pivotal, underappreciated element in the complex immune landscape of breakthrough infection is the eosinophil. Traditionally aligned with type 2 immunity, eosinophils have been tied to both protective and pathological facets of the host response to respiratory viruses. There are many points in the cascade of events that ultimately result in breakthrough infection of vaccinated hosts upon subsequent exposure to the target respiratory virus where eosinophils could play a role.

### 3.1 Protective and pathogenic roles for eosinophils during vaccination

The role of eosinophils during primary antigenic exposure, in this case vaccination, could be important for later secondary antigenic exposure to the target virus during infection. Beginning with the act of intramuscular vaccination itself, eosinophils have been shown to infiltrate injured skeletal muscle to mediate repair alongside alternatively activated macrophages (M2) by secreting IL-4 and stimulating fibroadipogenic progenitors, and eosinophil-deficient ΔdblGATA-1 mice show impaired regeneration.^98^ Beyond muscle injury, multiple groups have also demonstrated that vaccination, particularly in the presence of adjuvant, can recruit eosinophils to the injection site.^99^ Although eosinophils were not specifically quantified, CD11b^+^Gr-1^+^ granulocytes were recruited to the muscle injection site in 16 to 72 h by type 1– and type 2–skewing adjuvants.^100^ Histopathological examination has demonstrated the recruitment of eosinophils at the injection site as early as 3 to 6 h in the muscle after administration of alum, a well-documented type 2–skewing adjuvant.^101,102^ High numbers of eosinophils can be observed at 26 h postvaccination intraperitoneally, with rapid local expression of IL-5 peaking at 5 h postinjection.^103–105^ AS03, an adjuvant composed of α-tocopherol and squalene in an oil-in-water emulsion capable of eliciting both a Th1 and Th2 response, induced greater recruitment of eosinophils than alum to the muscle-draining lymph node 24 h postinjection, alongside higher CCL11 protein levels in the muscle and mRNA expression of eosinophil-recruiting chemokines CCL5, CCL11, and CCL24 in the draining lymph node.^106^ Vaccination of mice with ovalbumin (OVA) adjuvanted with MF59 (an emulsion of 5% squalene, 0.5% TWEEN 80, 0.5% Span 85 in citrate buffer) resulted in detectable eosinophil recruitment in the muscle beginning at 16 h postinjection that peaked at 48 h, then gradually decreased through the study endpoint of 20 d postinjection.^107^ Together, these data suggest that oil-in-water emulsion adjuvants and alum are capable of recruiting eosinophils to the muscle after injection. It is not yet established if the recruitment of eosinophils to adjuvanted vaccine injection sites in the muscle also occurs with potent type 1–biasing, toll-like receptor (TLR) agonist adjuvants that are commonly used in vaccines for respiratory viruses, such as poly(I:C) or glucopyranosyl lipid adjuvant-stable emulsion (GLA-SE), although TLR expression and activity are well documented for both mouse and human eosinophils.^108–112^

**Fig. 1.**
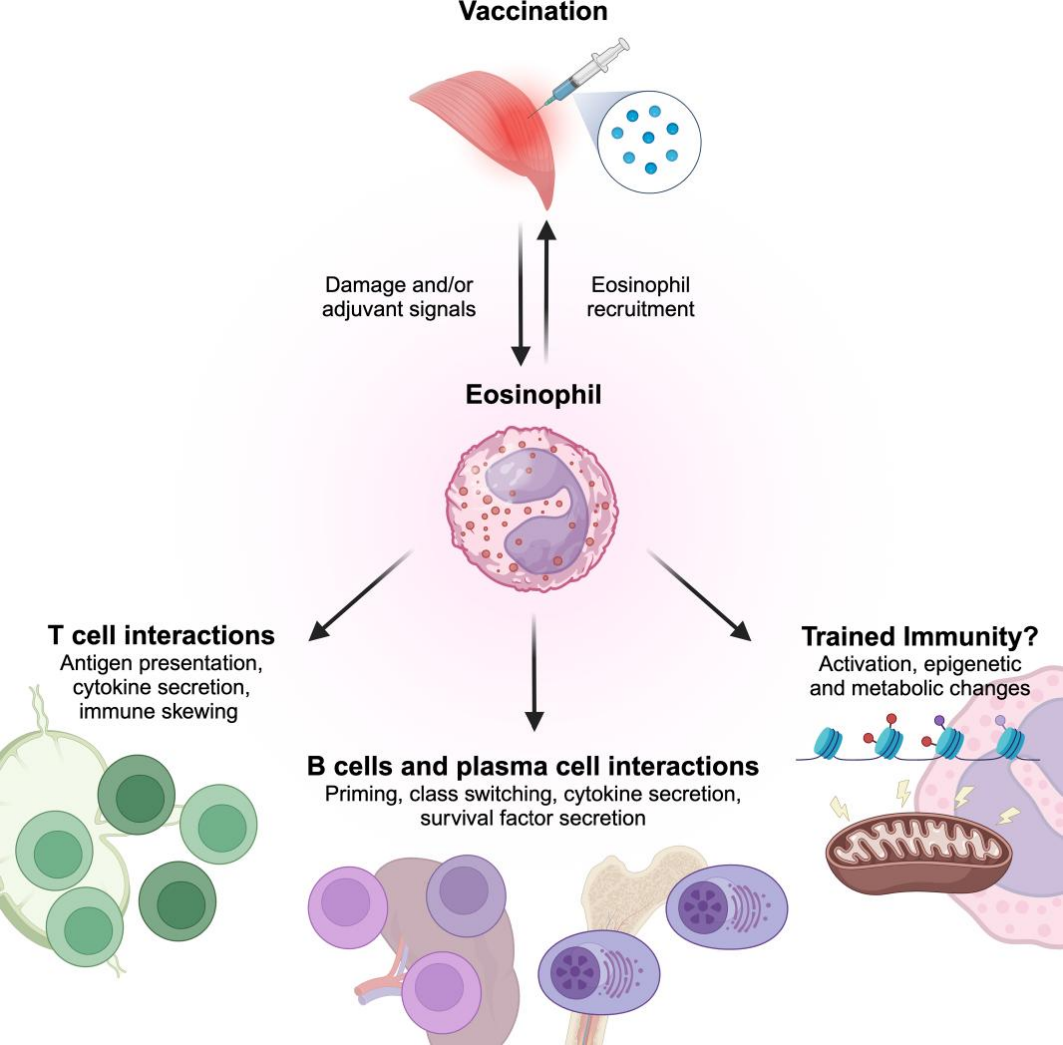
Putative roles for eosinophils after vaccination. Shortly after vaccination, eosinophils are recruited to the injection site, usually skeletal muscle for intramuscular vaccination, by muscle damage and adjuvant signals. Eosinophils can then (1) traffic to the lymph node, present antigen to T cells, and then secrete cytokines such as IL-4 and IFN-γ to polarize T cells toward Th1 or Th2 phenotypes; (2) traffic to the spleen to prime B cells and promote class switching through cytokine secretion, or traffic to the bone marrow to secrete survival factors to promote plasma cell maintenance; and (3) undergo cell-intrinsic changes such as trained immunity in response to vaccine and adjuvant stimuli, resulting in epigenetic and metabolic alterations in existing eosinophils or bone marrow progenitors to promote a more activated state and enhanced responsiveness against inflammatory insults.

The presence and recruitment of eosinophils at the vaccine injection site has several implications for subsequent vaccine-derived immunity (Fig. 1). First, eosinophils are capable of trafficking to lymph nodes and antigen presentation to T cells.^59,113–116^ Therefore, eosinophil recruitment to the site of vaccination by both muscle damage and adjuvants and subsequent polarization by adjuvant-intrinsic stimulatory effects could contribute to T-cell skewing and downstream immunity upon secondary antigen encounter. For instance, in the context of allergy, eosinophil-derived IL-4 release during antigen presentation skews CD4 T cells toward the Th2 phenotype.^117,118^ After vaccination, it is possible that eosinophils may aid in the skewing of host responses toward type 2 due to secreting IL-4 and polarizing CD4 T cells toward Th2, similar to what was seen in allergy. Overt polarization of T-cell responses postvaccination toward Th2 for respiratory viral vaccines, like in the case of FI-RSV, may result in worse host outcomes.^33^ It has been suggested that eosinophils could shift host responses toward type 1 after vaccination. Human blood eosinophils are capable of producing type 1 cytokine IFN-γ in response to ex vivo stimulation with rhinovirus. This suggests that appropriate adjuvant selection could allow skewing of T cells toward Th1 or balanced Th1/Th2 phenotypes as mediated by eosinophil cytokines, which would then be more beneficial for antiviral immunity.^58,119^ However, eosinophil-deficient mice and humans undergoing eosinophil-depleting treatments appear to have intact, protective responses to vaccination; thus, eosinophils are likely not the major driver for T-cell priming.^33,120–122^ The extent of eosinophil contribution to vaccine-elicited T-cell priming remains undefined. It is important to note that findings in eosinophil-deficient ΔdblGATA-1 mice may not be entirely attributable to the reduction in eosinophils since a more widespread immunodeficiency in myeloid cell development has been described by Hwang et al.,^123^ including a global reduction in CD11b^+^ cells, such as inflammatory monocytes and monocyte-derived dendritic cells (DCs), and reductions in antigen presentation-related gene expression in monocytes. GATA1 knockout of CD11c^+^ cells, such as DCs, was previously shown to impact DC migration toward CCL21, and this may also be at play in ΔdblGATA-1 mice.^124^ Thus, antigen presentation to and priming of lymphocytes may be impaired in ΔdblGATA-1 mice not solely because of eosinophil deficiencies but also because of functional and numerical deficiencies in critical antigen-presenting cell (APC) populations.^125–127^

Second, eosinophils can migrate to the bone marrow and spleen, where they interact with B cells and long-lived plasma cells (LLPCs) that maintain immune memory and protective levels of neutralizing antibodies in circulation long after vaccination.^128,129^ Alum-adjuvanted intraperitoneal immunization can induce IL-4^+^ eosinophil migration to the spleen and prime B cells to secrete antigen-specific IgM, indicating their potential to kickstart vaccine-specific antibody responses.^128^ It is possible that eosinophil-derived IL-4 could promote IgE class switching in B cells, generating vaccine-specific IgE antibodies and promoting allergic responses to vaccine antigens.^130,131^ However, it is unlikely that this is a major concern since vaccine allergies are very rare and multidose vaccines administered with type 2 adjuvants do not have a significantly higher rate of vaccine-elicited allergic reactions.^132–135^ Instead, most vaccine-associated allergic adverse events are in individuals with preexisting allergies to vaccine components, such as egg proteins.^132–135^ LLPCs reside in the bone marrow and stably secrete antibody for long periods of time, sustaining vaccine-elicited humoral protection.^136–138^ Within the bone marrow, eosinophils can secrete cell survival factors like IL-6 and APRIL to support plasma cell maintenance, although how necessary these cells are for this process is contested.^120,139,140^ Additionally, there was no significant difference in the titer and longevity of antigen-specific IgG in wild-type BALB/c mice vs eosinophil-deficient ΔdblGATA-1 mice through 70 d after intraperitoneal vaccination, so eosinophils may not be majorly influential in shaping the overall quantity of antibody.^140^ Furthermore, neither eosinophils nor neutrophils were required for the maintenance of nasal tissue Sendai virus–specific IgG^+^ or IgA^+^ antibody-secreting cells following intranasal challenge.^141^ Investigating the impact of eosinophils on antibody subtype, gene usage, and somatic hypermutation rates could be of interest. Of note, many of these studies involved peritoneal injection of mice, whereas most human immunizations are intramuscular.^142^ Thus, the specific contribution of muscle vs peritoneal eosinophils on the quantity and quality of vaccine-elicited antibodies, resultant isotypes, neutralization, and longevity of the humoral response remains to be elucidated for viral vaccines and antigens.

Third, muscle or injection site eosinophils could be exhibiting trained immunity.^143–145^ Similar to how adaptive immunity has memory, trained immunity is “de facto innate immune memory”: innate immune cells that have recently experienced inflammatory insults such as vaccination or infection are functionally reprogrammed via metabolic and epigenetic changes to have heightened responsiveness to homologous or heterologous insults.^143^ Vaccination with the Bacillus Calmette-Guérin (BCG), measles-mumps-Rubella (MMR), influenza, and oral polio vaccines (OPVs) can confer some degree of heterologous protection beyond the target disease in an antigen-agnostic manner.^146^ For example, vaccination of healthy adults with a seasonal trivalent inactivated influenza vaccine (TIV) or an AS03-adjuvanted inactivated split-virion H5N1 vaccine resulted in enhanced antiviral capabilities in blood myeloid cells, particularly monocytes, as well as resistance ex vivo against dengue or Zika virus infection.^147^ Although eosinophils have not been the main focus of many vaccination-induced trained immunity studies, Moorlag et al.^148^ tangentially found that BCG vaccination of BCG-naive healthy adults resulted in higher levels of activation marker CD66b and reduced PD-L1 expression on the surface of eosinophils postvaccination compared to prevaccination, with no changes in eosinophil count in the blood. The consequences or benefits of increased eosinophil activation and PD-L1 downregulation were not investigated but could potentially have implications on subsequent heterologous inflammation. Of note, eosinophils have a short half-life in most tissues, which begs the question of how relevant trained immunity could be during a breakthrough infection occurring many months after the priming vaccination.^50^ It is possible that similar to neutrophils, which also exhibit a short half-life, eosinophil progenitors in the bone marrow are the ones trained instead, allowing for more long-term persistence of trained immunity.^149^ At the time of writing, there are no studies specifically assessing trained immunity in eosinophils and their bone marrow progenitors after vaccination, or their role in mediating heterologous protection from secondary inflammatory insults in sites distal from priming, such as in the respiratory tract. We look forward to new insights in the field as technical limitations are gradually overcome with constantly improving single-cell technologies capable of producing high-quality data for fragile and ribonuclease (RNAse)-rich cells like eosinophils. Understanding peripheral training of eosinophils and how they are functionally remodeled, not just after vaccination, is relevant for the fields of allergy, asthma, and other eosinophilic diseases as well. It will be important to investigate how strongly vaccination induces trained immunity in eosinophils and their bone marrow progenitors, if it can be modulated by the inclusion of adjuvants, if vaccine-trained eosinophils are recruited to the lung during breakthrough infection, and if their phenotype and effector functions are altered by training.

In summary, eosinophils can impact subsequent adaptive and innate immune responses in the context of vaccination in a multipronged fashion. Eosinophils are recruited to the injection site during vaccination and play a role in tissue repair and regeneration; as such, they are in the right location during a primary antigenic exposure to exert influence on vaccine immunity. Adjuvants like alum and oil-in-water emulsions induce eosinophil recruitment, but the response to type 1–skewing adjuvants remains underexplored in vaccination scenarios. Eosinophils present at the injection site have implications for subsequent vaccine-derived immunity, potentially contributing to T-cell priming and skewing due to their ability to traffic to draining lymph nodes and present antigen. While eosinophils have been shown to interact with B cells and LLPCs, their specific impact on the quantity and quality of vaccine-elicited antibodies and humoral responses remains uncertain. The impact of trained immunity in eosinophils and how it may modulate antigen-agnostic immune responses is a topic warranting further investigation with evolving single-cell technologies. Regardless, the impact of eosinophil–lymphocyte interactions on vaccine immunity is likely outweighed by the contribution of the canonical professional APC–lymphocyte interactions. The field would benefit from more mechanistic studies using conditional knockout models such as the iPHIL mice to disentangle eosinophil contributions from potentially unwanted, confounding defects in the APC population present in other mouse models.^121,123,150^ Given phenotypic and transcriptional differences between gastrointestinal eosinophils from lung eosinophils, functional comparison of how muscle vs peritoneal or skin eosinophils differentially impact vaccine responses could augment our understanding on the importance of injection site innate immunity.^151^

### 3.2 Protective and pathogenic roles of eosinophils during breakthrough infection

Eosinophils have been shown to promote host resistance to a wide variety of pathogens, such as bacteria, fungi, and parasites.^152–163^ The many functions of eosinophils during respiratory viral infections have been thoroughly reviewed and well studied in the context of naive hosts undergoing primary infection in preclinical animal models.^58,164–168^ Our interest lies in the role of eosinophils in hosts with preexisting immunity conferred by vaccination, with a focus on lung eosinophils.

#### 3.2.1 Currently known instances of lung eosinophilia following breakthrough infection

Lung eosinophilia after breakthrough infection generally has a negative connotation due to its strong association with VAERD and pathology, such as for FI-RSV.^30,33,36,64^ VAERD in humans and animal models has been reviewed in depth by Bigay et al.^35^ and Munoz et al.^34^ For FI-RSV, the mechanism of VAERD after RSV infection has been thoroughly investigated and can be linked to the combination of Th2-biased CD4 T cells and induction of weak to nonneutralizing antibodies.^169,170^ Beyond FI-RSV, other vaccine–virus pairs have been observed to elicit lung eosinophilia upon subsequent viral challenge as well, although the degree of pathology and negative host outcomes varies in mouse models.

Here we highlight a noncomprehensive selection of instances where lung eosinophilia upon breakthrough infection resulted in worse, pathological outcomes. Adult BALB/c mice vaccinated with ultraviolet (UV)–inactivated whole SARS-CoV virion were not protected from lethal challenge or lung viral replication and exhibited significant lung eosinophilia as well as high levels of IL-4 and IL-13.^38^ Some mice exhibiting lung eosinophilia died after viral challenge, potentially due to the aberrant inflammation, but in the absence of positive immunohistochemical staining for SARS-CoV antigen in the lung.^38^ Adjuvanting the vaccine with TLR agonists prevented eosinophilia and expression of IL-4, IL-13, and eotaxin in the lung.^38^ Similar to findings in younger mice, aged BALB/c mice vaccinated with whole SARS-CoV virion, double-inactivated with UV irradiation and formalin, resulted in enhanced type 2 cytokine mRNA expression in the lungs, enhanced pathology, and lung eosinophilia but did not correlate with weight loss or mortality.^37^ Beyond inactivated vaccines, VAERD was also observed with protein vaccination. BALB/c mice vaccinated with the ectodomain of the SARS-CoV-2 spike (S) protein with or without alum resulted in suboptimal neutralizing antibody titers, type 2–biased immune responses, and disease enhancement with eosinophilic lung immunopathology, which could be abrogated when vaccine was adjuvanted with TLR agonists instead.^171^ Passive immunization of high neutralization capacity serum in vaccinated mice did not prevent eosinophilic infiltration, nor did transfer of vaccinated mouse serum into naive mice induce eosinophilia, pointing toward T cells or other immune cells as drivers of the VAERD phenotype rather than antibody in this model.^171^ Both mRNA and viral vector vaccines are thought to induce strong type 1 immunity and have also been evaluated for their potential to induce VAERD. In a study directly comparing mRNA vaccines with known VAERD-inducing vaccines, BALB/c mice were vaccinated in a prime-boost regimen with whole inactivated SARS-CoV with alum, heat-denatured SARS-CoV-2 S protein with alum, or mRNA vaccine encoding SARS-CoV-2 S (mRNA-1273).^172^ All vaccinated mice were protected from substantial weight loss upon challenge with mouse-adapted SARS-CoV-2, but positive staining for eosinophil major basic protein (MBP) was observed in the lungs at 4 d postinfection in the non-mRNA vaccine groups, alongside induction of antigen-specific Th2 CD4 T cells, type 2 cytokines in the lung, and IgG1/IgG2a ratios skewed toward IgG1.^172^ Mice vaccinated with mRNA-1273 exhibited strong type 1 skewing for all metrics, control of viral replication, and no lung eosinophilia.^172^ Syrian golden hamsters vaccinated with measles virus–vectored SARS-CoV-2 S did not lose weight, had low pathology scores, had no eosinophilic influx in lung sections, had controlled viral replication, and exhibited strong type 1 but no type 2 cytokine secretion after challenge.^40^ Hamsters that received recombinant nonstabilized S protein with alum adjuvant had more weight loss, lung eosinophilia, minimal control of viral titers, and overall type 2 biased responses.^40^ Dexamethasone administration to the S protein with alum group did not prevent Th2 cytokine production but reduced lung pathology and eosinophil counts.^40^ Dexamethasone, a glucocorticoid, is used to treat asthmatic patients due to its ability to tamp down airway inflammation and lower eosinophil numbers via apoptosis.^173^ Influenza challenge of pigs vaccinated with whole-inactivated virus can also exhibit enhanced disease phenotypes, although the mechanism appears to be linked to nonneutralizing antibodies rather than type 2–biased vaccine memory.^42,43,174,175^ Overall, it appears that inappropriate skewing of host vaccine immunity toward type 2 responses drives antigen-specific Th2 CD4 T-cell formation, which, upon restimulation with viral antigens during challenge, secretes large amounts of IL-4, IL-5, and IL-13. This in turn promotes lung eosinophilia, mucus hypersecretion, and airway hyperresponsiveness, leading to worse host outcomes.

There are also instances when lung eosinophilia is observed but without the same degree of enhanced disease as in VAERD. Mice vaccinated with formalin-inactivated pneumonia virus of mice (PVM) antigens and then challenged with PVM had prominent Th2 cytokines in the bronchoalveolar lavage fluid (BALF), lung pathology, and no detectable neutralizing antibodies in the serum, yet exhibited a 10-fold reduction in viral titer and reduced weight loss compared to mock-vaccinated mice.^176^ While there are multiple examples of VAERD induced by inactivated SARS-CoV and SARS-CoV-2 vaccines, a study with another coronavirus, Middle East respiratory syndrome coronavirus (MERS-CoV), showed protection instead. Vaccination with gamma-irradiated whole-inactivated MERS-CoV adjuvanted with alum or MF59 exhibited protection despite lung eosinophilia.^177^ All vaccine-recipient mice generated high neutralizing antibody titers and substantially lower viral titers than adjuvant-only controls, but lung histopathology analyses revealed moderate levels of eosinophilic infiltration at 6 d postinfection with 100 LD_50_ of MERS-CoV alongside an increase in lung IFN-γ, IL-5, and IL-13.^177^ For influenza, a BALB/c model of nonsterilizing, sublethal infection of mice vaccinated with TIV demonstrated lung eosinophil influx via flow cytometry at 7 d postinfection, but this was correlated with protection and not worse outcomes since vaccinated hosts exhibiting lung eosinophilia had less weight loss than unvaccinated control mice, controlled viral titers, hemagglutination inhibition titers, nonallergic serum IgE levels, and no strong type 2 cytokine signal.^44–46^ Furthermore, inclusion of a TLR agonist adjuvant shifted the IgG1/IgG2a ratio toward IgG2a but did not prevent lung eosinophilia.^46^

Many studies highlighted here were conducted in BALB/c mice, which are thought to be type 2 biased compared to C57BL/6 mice due to genetic differences in the immune system.^178,179^ It is possible that strain-specific immune biases drive systemic type 2 responses and thereby promote lung eosinophil recruitment in response to viral challenge after vaccination in BALB/c mice. However, in the context of allergy, it appears that C57BL/6 mice are more prone to generating Th2 responses in the lung while BALB/c mice generate Th1 responses, despite high levels of IL-4 and IL-5 produced by BALB/c splenocytes.^180^ The impact of strain genetic background on local and systemic immunity during breakthrough infection should be investigated further.

When lung eosinophils are associated with pathological outcomes, eosinophilia is severe and immediately apparent in histopathology, with minimal improvement in control of viral replication in vaccinated groups and generally accompanied by robust type 2 cytokine signals in the lung. In contrast, when lung eosinophils appear but do not result in overtly pathological outcomes, there is evidence of some degree of viral inhibition, although these outcomes are highly heterogeneous, with no clear unifying factor other than the use of inactivated virus vaccine in the highlighted examples. There are fewer studies describing lung eosinophilia correlating with viral protection in the context of vaccination challenge experiments than those describing enhanced disease, limiting our ability to identify other commonalities in the etiology of nonpathological lung eosinophils during secondary antigenic exposure.

Of note, the limited data availability on lung eosinophils may be largely due to technical limitations of standard methods, including the omission of eosinophil markers from flow cytometry panels, lack of eosinophil-specific gating in flow cytometry analyses, and the ability to resolve subtle increases in eosinophils via histopathology evaluation. It is possible that other preclinical animal studies investigating vaccination challenge regimens for respiratory viruses elicited lung eosinophils that were not overtly apparent or quantified in histopathological evaluation, or eosinophil markers or gates simply were not included in flow cytometric assessment of lung immune cells and therefore not measured at all. For example, our group did not notice the presence of eosinophils in our breakthrough influenza infection lung samples during manual gating, until we applied unbiased, unsupervised clustering analysis to our flow cytometry data. Thus, preclinical evaluation of vaccines should include some degree of lung myeloid quantification alongside pathology assessment during challenge studies in case an eosinophil signal is present. Whether or not nonpathological lung eosinophilia occurs during breakthrough infection in humans remains unknown. Only lung samples from deceased patients following virus infection are typically analyzed in depth for aberrant host immune responses to infection, sometimes characterized by eosinophilia, and are therefore biased toward cases with severe disease outcomes. Acquiring lung or BALF samples from human cases of breakthrough infection in vaccinated hosts also poses many difficulties, including the sampling techniques themselves and ensuring sample timing is within the same relative window after symptom onset or test-confirmed positive result for all breakthrough cases. Perhaps existing cohorts or protocols tracking vaccine and illness immune metrics that collect both blood and mucosal samples could be leveraged by measuring eosinophil counts, tracking the quantity of eosinophil granule products as a surrogate for degranulation, then correlating values with viral load measurements, for example. These could be additional measurements included as clinical trial endpoints.

#### 3.2.2 Functional role of lung eosinophils during breakthrough infections

**Fig. 2.**
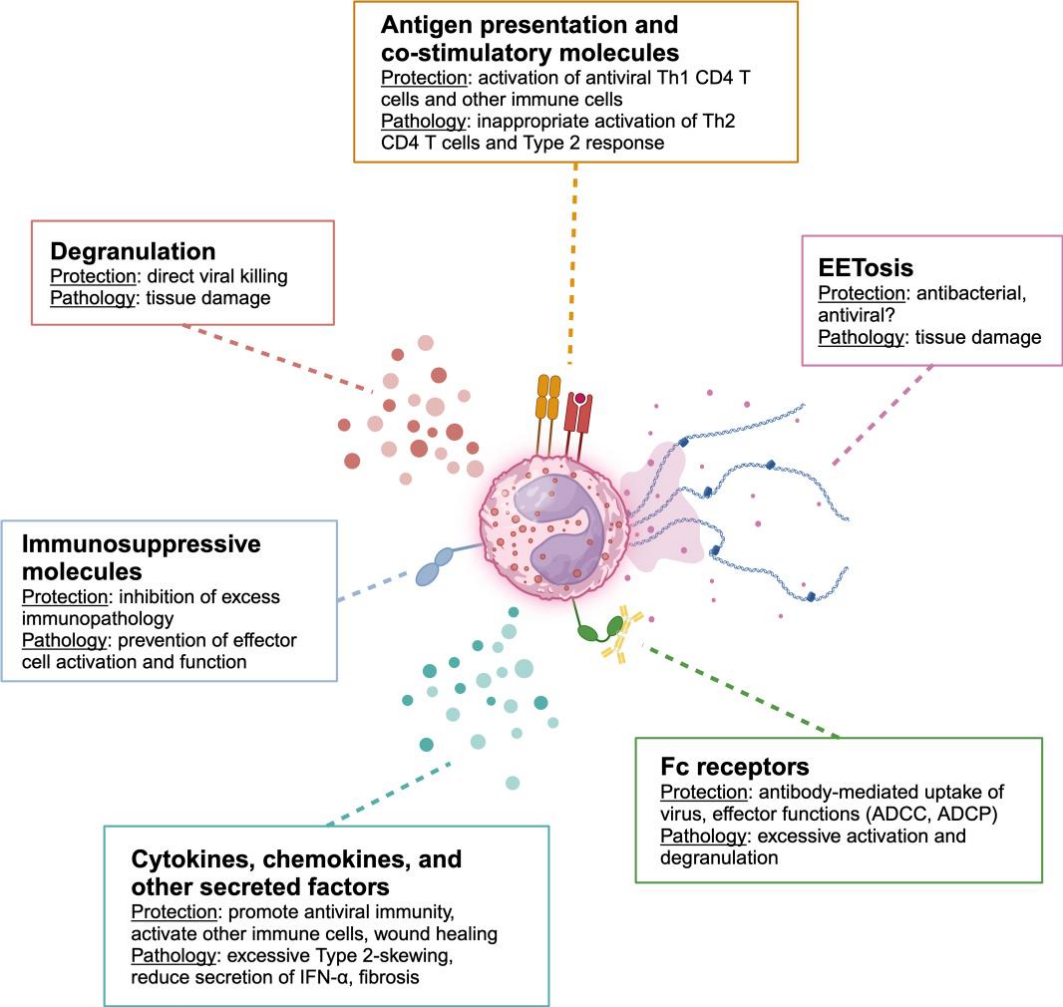
Putative roles for eosinophils after breakthrough infection. Eosinophils have a wide arsenal of tools to combat viral infection that have been described for primary infection but likely are protective during breakthrough infection: antigen presentation to T cells in the infected tissue, activation of other immune cells via costimulatory molecules, EETosis, Fc receptor engagement, cytokines and other secreted factors, immunosuppressive molecules, and degranulation. Each of these functions can confer protection against viral infection or result in pathology in vaccinated hosts.

During breakthrough infection, there are multiple direct and indirect mechanisms that eosinophils could be contributing to in the host immune landscape (Fig. 2). The antiviral capabilities of eosinophils during primary infection have been extensively reviewed, and while it is not the major focus of this review, we will briefly highlight some examples where the eosinophil response to primary infection may be applicable to investigations into breakthrough infection.^58,164–168^

One of the mechanisms by which eosinophils can mediate damage during breakthrough infection, either to the host or to the virus, is through degranulation. Eosinophils can undergo piecemeal degranulation (PMD), exocytosis, or cytolysis, each with distinct regulatory and driving mechanisms. PMD involves gradual release of cytoplasmic secretory granule contents through vesicular transport.^181^ Exocytosis is the release of granular or vesicular contents into the extracellular space.^181^ This release occurs either through the fusion of granules directly with the plasma membrane, known as classical exocytosis, or through the fusion of intracellular granules with each other before interacting with the plasma membrane, a process termed compound exocytosis.^181^ Cytolysis, on the other hand, involves release of whole granules and cytoplasmic contents immediately after plasma membrane rupture during rapid, nonapoptotic cell death.^181^ Degranulation is a double-edged sword: PMD can have direct antiviral effects and promote clearance of the infection in mouse models of PVM, RSV, and influenza but can also induce tissue damage, as demonstrated in human children for RSV.^59,64,182–186^ Understanding how to control the scope and type of eosinophil degranulation triggered may provide clues on how to maximize its effectiveness as an antiviral tool while limiting the tissue-damaging effects. How type 1 or type 2 polarization, conferred by vaccine priming of the host immune system, impacts the nature of eosinophil degranulation and its effects on other cells in vivo has not been thoroughly explored.

Eosinophils are capable of producing extracellular traps (EETs), similar to neutrophil extracellular traps (NETs), characterized by release of DNA studded with eosinophil granule proteins and histones.^187^ EETs have been demonstrated to have antibacterial properties and can also be released in response to viruses such as RSV.^187–189^ The antiviral activity of NETs has been described but has yet to be directly demonstrated for EETs.^190^ A pathogenic role for extracellular histones, as found on EETs and NETs, has been described in a mouse model for influenza challenge, with exacerbated lung pathology and injury.^191^ When considering host outcomes, the antipathogen features of ETs need to be balanced with the potential for enhanced pathology and tissue damage. A better understanding of what controls ET release and the scale of release, as well as if training of granulocytes or their progenitors after vaccination has an impact on these mechanisms, can allow for more careful adjuvant selection and vaccine design to leverage the virucidal effects of ET deployment during secondary viral encounter while reducing unwanted lung damage.

There is some evidence that eosinophils can contribute to antiviral immunity through direct engulfment of viral particles, which may be dampened in a type 2–biased environment such as asthma.^166,192,193^ Eosinophils also express Fc receptors (FcRs) and can bind to virus–antibody immune complexes as another mechanism of reducing viral load.^113,194^ It is possible that during breakthrough infection, eosinophils could bind to vaccine-derived antibodies via FcR and engage in effector functions like antibody-dependent cellular cytotoxicity (ADCC) or phagocytosis (ADCP) to help clear virus.^195–197^ Binding of secretory IgA to eosinophils can induce degranulation, which could be beneficial for clearance of virus or result in tissue damage.^198^ Eosinophils are capable of internalizing and processing antigens for MHC II presentation and can upregulate MHC I in response to direct viral infection with influenza.^59,113^ Costimulatory molecules such as CD28, CD40, CD80, and CD86 have been measured on the cell surface of eosinophils.^58,114^ In conjunction with their antigen presentation capabilities, costimulatory molecule expression, and cytokine secretion, eosinophils could have an impact in dictating the nature of the secondary immune response in a vaccinated host to virus by engaging with memory T cells and skewing them either toward Th1 or Th2 phenotypes, or both. Eosinophils could also contribute to mucosal immunity through participating in bronchus-associated lymphoid tissue (BALT) formation. Although not an example from viral infection, eosinophil-deficient ΔdblGATA-1 mice had reduced proinflammatory cytokine concentrations, impaired lymphocyte recruitment, and deficiencies in BALT during *Bordetella bronchiseptica* infection of the lungs.^199^ BALT is an important site for generating local mucosal immunity and can enable more rapid control of respiratory infection, and the need for effective BALT formation for eosinophils poses another area of exploration for intranasal vaccine development.^200^ Again, it is possible defects in other myeloid cells like DCs, which are critical for BALT formation after influenza infection, in the ΔdblGATA-1 mouse model are confounding the eosinophil-specific contributions.^123,200,201^ It is also likely that other APCs and phagocytes in the lung and airways outperform eosinophils at these functions, such as macrophages and DCs. However, the specific contribution of eosinophils toward helping or hindering antiviral immunity in vaccinated hosts has not been precisely dissected yet and could have major implications in hosts with preexisting high numbers of lung eosinophils, such as asthmatics.

Eosinophils can also produce and release a multitude of cytokines, chemokines, and growth factors.^202,203^ Cytokines can be found in preformed granules and released more rapidly compared to T cells, which have to undergo tightly regulated de novo synthesis to secrete cytokines.^202,203^ Steady-state human eosinophils have been shown to have preexisting stores of IFN-γ, TNF-α, IL-12 (p70), IL-4, IL-13, IL-6, and IL-10 that can be differentially released depending on the type of stimulus.^204,205^ Given that eosinophils can be found in the lung at steady state and can rapidly secrete cytokines before peripherally induced vaccine-specific memory CD4 T cells can maximally infiltrate infected tissue, there is potential for eosinophils to act as early orchestrators of the innate immune response during viral infection through release of immune-polarizing cytokines or growth factors for other cells.^206^ Even if eosinophils are not the major cytokine-secreting cells in the lung, they have been shown to alter the function of other immune cells that more strongly shape the cytokine milieu, such as plasmacytoid DCs (pDCs). Coculture with eosinophils or eosinophil supernatants inhibited the ability of human blood–derived pDCs to secrete IFN-α, critical inducers of the host antiviral state, in response to rhinovirus 16 in an ex vivo cell culture model.^207^ This effect was partially attributable to secretion of eosinophil-derived neurotoxin (EDN) and TGF-β by eosinophils.^207^ Eosinophil-derived TGF-β has also been implicated in wound healing, nasal polyp formation, and lung fibrosis, again demonstrating both beneficial and detrimental roles for these cells depending on contextual cues.^49,98,208^ In influenza infection of mice, eosinophils accumulate in the lung after viral clearance during the recovery phase at days 12 to 16 postinfection, but their necessity for postinfection recovery has yet to be determined.^209^ There is a need for further investigations into the specific contribution and magnitude of influence that eosinophil-derived cytokines have on the outcomes of breakthrough infection.

Another function eosinophils could be exerting during breakthrough infection is immunosuppression. As discussed, eosinophils are capable of secreting IL-10, broadly thought to be an immunosuppressive cytokine. Eosinophils, particularly those that have been stimulated with type 1–skewing agents such as lipopolysaccharide and IFN-γ, can upregulate surface expression of PD-L1.^151,210,211^ PD-L1 binding to PD-1 on T cells counterbalances activation, allowing for a balance between cytotoxic destruction of infected cells and maintenance of tissue integrity.^212^ PD-L1 can bind to costimulatory, activating molecule CD80 on immune cells in an inhibitory manner as well.^212^ Insufficient PD-1 signaling can result in T-mediated immunopathology during acute viral infections.^212^ Eosinophil suppression of inflammation via PD-L1 has been demonstrated for other models but not yet in primary or breakthrough respiratory viral infection. For example, gastrointestinal eosinophils are capable of suppressing Th1/Th17 responses during *Helicobacter pylori* infection through IFN-γ–dependent upregulation of PD-L1.^213^ Similarly, IFN-γ–polarized eosinophils can produce inducible nitric oxide synthase (iNOS) to facilitate nitric oxide (NO) secretion, upregulate PD-L1 to synapse with T cells, dampen T-cell receptor signaling, and prevent allograft-rejecting CD8 T-cell effector activity in a mouse lung allograft model.^214^ Surface expression of CD101, another putative immunosuppressive molecule, has also been reported for both human and mouse eosinophils and appears to be on a subset that predominantly localizes to the peribronchial or perivascular regions, although the CD101^+^ eosinophil subset is thought to be proinflammatory.^215,216^ CD101 has been used as a marker of mature neutrophils but has also been described for other myeloid cells as well, such as dendritic cells.^217–219^ The function of or binding partner to CD101 is not known, especially on eosinophils, but immunosuppressive properties have been described for other myeloid cells. Antibody stimulation of CD101 on cutaneous DCs conferred the ability to inhibit T-cell proliferation, with concomitant upregulation of IL-10 secretion.^220^ In the context of a colitis mouse model, the presence of CD101^+^CD11b^+^ myeloid cells was required for optimal Treg immunosuppression of colitogenic T cells.^221^ CD101^+^ eosinophils were enriched for in mice undergoing influenza breakthrough infection and correlated with improved host outcomes.^46^ Given its association with immunosuppression, it will be interesting to see if CD101 confers inhibitory ability to eosinophils and prevents immunopathology during viral infection in future investigations. Eosinophil-specific ablation of CD101 could address the necessity of this surface molecule and cell phenotype, as well as how the absence of CD101^+^ eosinophils impacts inflammation.

It is also possible that eosinophils themselves are not essential mediators of pathology or protection during breakthrough infection, but lung eosinophilia is a mere consequence of type 2–biased immunity conferred during vaccination. For example, in models of FI-RSV enhanced disease, use of ΔdblGATA-1 eosinophil-deficient mice has shown that eosinophils themselves are not the major drivers of disease.^33,222^ Rather, RSV-specific Th2-biased immune responses were required for pathological metrics such as mucus hypersecretion and airway hypersensitivity, while type 1 cytokine TNF-α was a critical driver of airway obstruction and weight loss.^33^ A study in cotton rats, a more permissive host for RSV infection, vaccination with the original Lot 100 FI-RSV followed by RSV challenge found that eosinophils are actually a very small proportion of the lung cellular infiltrate after challenge.^32^ This brings into question if such a small population of cells is sufficient to drive the widespread host pathological effects.^32^ Still, while eosinophils may not be directly implicated in driving the disease phenotype in VAERD, it is possible that recruited eosinophils drive damage or protection for other vaccine–virus pairs. Nevertheless, more mechanistic studies of eosinophils in breakthrough infection scenarios, incorporating depletion or knockout models, to understand why they are recruited and how they are contributing to the immune environment, are required.

## 4. Balancing vaccine efficacy with eosinophil-related risks or benefits

From vaccination to primary infection or breakthrough infection, it is evident that eosinophils are pleiotropic cells that behave in both protective or pathogenic roles depending on the immune context. In vaccinated hosts, it is hard to fully discern the weight of eosinophil contributions to subsequent immunity, especially when other cell types such as dendritic cells have an outsized influence on the formation and skewing of vaccine memory. How necessary are eosinophils for all of these processes? We can partially answer these questions through studies done in eosinophil-deficient mouse models and patients undergoing eosinophil-reducing therapies.

### 4.1 Impact of eosinophil depletion on responses to vaccination

Vaccine responses, particularly with multiple different types of adjuvants, have not been evaluated in eosinophil-deficient mouse models yet. Models that do not have major defects in other APC populations, such as the MBP-1^−/−^ eosinophil peroxidase (EPX)^−/−^ mice, should be considered to minimize confounding factors.^150^ Although underexplored in mice, the impact of blood eosinophil-lowering drugs, such as monoclonal antibody benralizumab, on human vaccine responses has been evaluated.^223^ Serum hemagglutination inhibition and microneutralization titers were not significantly different between asthmatic patients receiving benralizumab (anti–IL-5Rα) or placebo after seasonal influenza vaccination, suggesting depletion of IL-5Rα^+^ cells did not compromise the humoral immune response to the vaccine.^122^ Additionally, transient absence of eosinophils in an infant born to a mother on benralizumab therapy did not appear to impact antibody levels after pneumococcal conjugate vaccine administration, which fell within normal ranges.^224^ More studies evaluating the impact of dupilumab on vaccine responses have been conducted. Dupilumab is a monoclonal antibody targeting the shared IL-4/IL-13 receptor and acts more broadly on type 2 immune pathways, rather than directly lowering eosinophil counts.^225^ Postvaccination antibody titers to tetanus toxoid, reduced diphtheria toxoid, and acellular pertussis (Tdap) vaccine antigens were similar in atopic dermatitis (AD) patients receiving dupilumab or placebo.^226^ Furthermore, vaccine-specific IgE was reduced in dupilumab recipients.^226^ Ungar et al.^227,228^ observed that patients with moderate-to-severe AD on dupilumab did not have impaired IgG antibody or T-cell responses to mRNA SARS-CoV-2 vaccines, with similar decay rates across all treatment groups. In contrast, Runnstrom et al.^229^ found that patients with severe asthma or AD on biologics (benralizumab, mepolizumab, or dupilumab) had lower IgG titers to SARS-CoV-2 compared to healthy adults at 25 to 49 d after the second vaccination. However, comparison of sera from a similar patient cohort but not receiving biologics likely would have been a more appropriate control than healthy adults, since the comorbidities present may have also had an impact on humoral immunity.^229^ Thus far, it appears that reducing eosinophil and type 2 responses does not have a detrimental effect on the vaccine responses of patients with AD or asthma. Whether or not reducing blood eosinophil counts or blocking type 2 responses would have any effect in healthy individuals without a preexisting type 2–skewed comorbidity remains to be seen. Of note, rare opportunistic herpes zoster infection has been reported in patients on some biologics targeting type 2 inflammation (dupilumab, mepolizumab, and benralizumab), and package inserts for dupilumab, tezepelumab, and tralokinumab recommend against live vaccine administration due to lack of data.^230^ Although generally safe and well tolerated, more investigations on the impact of these biologics on host antiviral and vaccine immunity are warranted.

### 4.2 Impact of eosinophil depletion on responses to respiratory viral infection

Eosinophil-deficient mice have mostly been used in head-to-head comparisons of presensitized host responses to subsequent viral infection but not for directly evaluating if eosinophils are necessary during viral infection alone. After OVA sensitization and challenge followed by parainfluenza virus challenge, mice overexpressing IL-5 predisposed to lung eosinophilia (NJ.1726) had reduced parainfluenza viral RNA in the lung, whereas IL-5–overexpressing eosinophil-deficient mice (NJ.1726-PHIL) and eosinophil-deficient mice (PHIL) did not have the same control of virus, with similar RNA levels as the wild-type infected mice.^63^ This suggests that the presence of eosinophils rather than high IL-5 concentrations is linked to improved viral control in OVA-sensitized mice.^63^ Wild-type C57BL/6 mice and eosinophil-deficient ΔdblGATA-1 mice were repeatedly sensitized with fungal pathogen *Alternaria alternata* in the upper airways.^231^ However, both types of mice were not protected from influenza infection, suggesting eosinophil accumulation did not confer an antiviral benefit in this model.^231^ Gorski et al.^209^ claim that eosinophil-deficient PHIL mice did not have greater susceptibility to lethal influenza challenge or worse recovery as measured via weight loss and lung inflammation as compared to wild-type mice, but the data for these observations were not published. Thus, the effect of eosinophil reduction in preclinical models without prior sensitization remains to be seen in currently available data. In humans, blood eosinopenia was flagged as a correlate of disease severity for coronavirus disease 2019 (COVID-19), although evidence suggesting otherwise also exists.^167^ Multiple studies evaluating the use of eosinophil-lowering biologics and risk of severe COVID-19 did not see worse outcomes in patients taking biologics.^223^ Interestingly, patients with mild asthma receiving mepolizumab, an anti–IL-5 monoclonal antibody that reduces blood eosinophil counts, had a higher viral load at day 7 postinfection than patients receiving placebo in a human rhinovirus challenge study, despite having higher secretory IgA levels.^232,233^ Whether or not this means asthmatic mepolizumab-treated patients have worse control of rhinovirus infection and do not generate effective, neutralizing mucosal antibodies will require additional studies. Once again, longitudinal comparison of responses to respiratory viral infection in healthy individuals with or without eosinophil-depleting biologics would be needed to fully disentangle the impact of eosinophils on the outcome of infection.

Collectively, more investigation is needed before making the judgment call of whether or not eosinophils are dispensable for vaccine and viral immunity. Given the potential for eosinophils to mediate protection or pathology, or perhaps both, how do we best leverage their antiviral capabilities while downsizing their potential for tissue damage?

### 4.3 Balancing eosinophil protective and pathological features

The most straightforward approach to balancing eosinophil-derived protection and pathology is through vaccine design, platform selection, and adjuvant choice. A noticeable proportion of the vaccines that are thought to elicit VAERD and adverse type 2 outcomes upon viral infection are inactivated vaccines, with or without alum adjuvant. There appears to be some propensity for inactivated virus vaccines to generate type 2 responses, including and beyond the FI-RSV example. Whole-inactivated influenza virus vaccination in mice resulted in high splenocyte IL-4 production upon restimulation in vitro, an IgG1/IgG2a ratio skewed toward IgG1 indicative of Th2 bias, and poor cytotoxic T-cell induction, although subsequent viral challenge pushed immune metrics more toward Th1.^234^ This study's relevance to humans may be limited since split influenza vaccines are currently the standard of care vaccination for the prevention of severe seasonal influenza disease, not whole inactivated virions.^235^ However, the method used to split whole inactivated virions into smaller components can also have an effect on immune skewing, as seen during the 2000–2001 influenza season in Canada with differing responses to 2 inactivated split vaccines containing the same 3 viral components.^236^ Fluviral S/F1 (Shire Biologics) was split with sodium deoxycholate and resulted in stronger Th2 responses in both mice and humans, whereas Vaxigrip or Fluzone (Aventis Pasteur) was split with Triton X-100 and elicited a more balanced Th1/Th2 response.^236^ This was only evident when assessing the ratio of IL-4^+^/IFN-γ^+^ and IL-5^+^/IFN-γ^+^ secreting cells, since both vaccines had similar IgG1, IgG2a, and IgE titers and IgG1/IgG2a ratios.^236^ The large aggregates of virus in the Fluviral S/F1 vaccine due to inefficient detergent splitting were postulated to drive the differences in polarization toward type 2.^236^ Perhaps the propensity toward type 2 immune induction during priming with inactivated antigens, due to potential factors like antigen alteration during the inactivation process or aggregation like in the split influenza vaccine example, is what drives lung eosinophilia upon second antigen encounter in the lungs.^236–238^ Therefore, while inactivated split or whole virion vaccines are easier to manufacture and have favorable safety profiles, a wide range of adjuvants should be included in preclinical testing, particularly those that elicit type 1 or balanced type 1/type 2 immune profiles. Alum can promote IL-10 secretion and inhibition of memory Th1 CD4 T-cell formation, so its use should be carefully evaluated.^239^ Lung eosinophils, by both histopathological examination and flow cytometry, should also be enumerated during vaccination challenge experiments. Setting a hard limit for eosinophil infiltration into the lung could be a means of eliminating vaccine candidates with risk of inducing VAERD early on in preclinical testing, such excluding vaccines where eosinophils are ≥5% of the lung immune infiltrates postchallenge, as proposed by Su et al.^240^

Although parenteral vaccines were the main focus of this review, intranasal vaccination may also provide another way to balance the protective and pathological effects of eosinophils in the airways. Multiple intranasal vaccine candidates, especially those coadministered with type 1–skewing adjuvants, induced mucosal immunity and effective viral clearance without overt eosinophilia beyond and enhanced pathology in mouse models.^241–247^ How intranasal vaccines confer trained immunity to eosinophils already present in the lung, the contribution of lung eosinophils to priming of T and B cells in lung-draining lymph nodes, the role of eosinophils in lymphoid tissue formation in the airways, and if lung eosinophils are required for effective intranasal vaccine immunity remain to be investigated in the future.

Other avenues to mitigate eosinophil-mediated adverse events following vaccination include understanding if subsets exist and if a specific eosinophil subset or activation status drives adverse events following vaccination or infection.^248^ This can be achieved by more in-depth preclinical studies and phenotyping of eosinophils from patient blood routinely collected in vaccine clinical trials using mass or flow cytometry. If a particular activation state or subset is correlated with worse outcomes, more specific targeting therapies could be developed to reduce the pathogenic eosinophil numbers. Thus, a more in-depth understanding of eosinophil heterogeneity will be beneficial. Monitoring of blood and airway eosinophil populations during phase 3 clinical trials of vaccines for respiratory viruses, particularly after breakthrough infection, could greatly add to our understanding of these critical cells and their utility as biomarkers of vaccine efficacy or adverse events.

## 5. Concluding remarks

As our understanding of eosinophil biology continues to grow, and more vaccines for respiratory viruses enter development or clinical testing, it is pertinent to understand how eosinophils impact vaccine responses, efficacy, and immunity, particularly when viral infection occurs. Eosinophils can infiltrate the muscle directly after injection and interface with both arms of adaptive immunity through antigen presentation, cytokine secretion, or costimulatory molecules. While much is known about the antiviral properties of eosinophils, much investigation needs to be done to bridge mechanisms seen in naive hosts to hosts with preexisting immunity. Much of the association of eosinophils with vaccinated hosts is during breakthrough infection of FI-RSV vaccinated infants in the 1960s, which resulted in VAERD. However, there appear to be some instances when nonpathological lung eosinophilia is observed as well, the significance of which is not fully known yet. In this review, we speculated on the potential pleiotropic functions of eosinophils in vaccinated hosts during breakthrough infection, ranging from direct virucidal effects, shifting the lung microenvironment with cytokines, to immunosuppression. Ultimately, more mechanistic studies will be required to understand what swings the balance toward pathology vs protection and if lung eosinophils are necessary for these responses at all.

## Abbreviations

AD: atopic dermatitis
ADCC: antibody-dependent cellular cytotoxicity
ADCP: antibody-dependent cellular phagocytosis
APC: antigen-presenting cell
BALF: bronchoalveolar lavage fluid
BALT: bronchus-associated lymphoid tissue
BCG: Bacillus Calmette-Guérin
BECC: bacterial enzymatic combinatorial chemistry
COVID-19: coronavirus disease 2019
DC: dendritic cell
EDN: eosinophil-derived neurotoxin
EET: eosinophil extracellular trap
EPX: eosinophil peroxidase
F protein: RSV fusion protein
FcR: Fc receptor
FI-RSV: formalin-inactivated respiratory syncytial virus
GLA-SE: glucopyranosyl lipid adjuvant-stable emulsion
HA: hemagglutinin
HSV-2: herpes simplex virus 2
iNOS: inducible nitric oxide synthase
LLPC: long-lived plasma cell
MBP: major basic protein
MERS-CoV: Middle East respiratory syndrome coronavirus
MMR: measles-mumps-Rubella
NET: neutrophil extracellular trap
NO: nitric oxide
OPV: oral polio vaccine
OVA: ovalbumin
pDC: plasmacytoid dendritic cell
PHAD: phosphorylated hexa-acyl disaccharide
PMD: piecemeal degranulation
PVM: pneumonia virus of mice
RSV: respiratory syncytial virus
S protein: coronavirus spike protein
SARS-CoV: severe acute respiratory syndrome coronavirus
SARS-CoV-2: severe acute respiratory syndrome coronavirus 2
Tdap: tetanus toxoid, reduced diphtheria toxoid, and acellular pertussis
Th1: T helper 1
Th2: T helper 2
TIV: trivalent inactivated influenza vaccine
TLR: toll-like receptor, Treg, regulatory T cells
UV: ultraviolet
VAERD: vaccine-associated enhanced respiratory disease
